# Supplementary material for: Energy Metabolism Disturbances in Cell Models of PARK2 CNV Carriers with ADHD
Source: J Clin Med. 2020 Dec 18;9(12):4092. doi: 10.3390/jcm9124092 (PMC7766864; doi:10.3390/jcm9124092)
Supplement: Supplementary file 1 [file jcm-09-04092-s001.zip › jcm-991097-supplementary/jcm-991097-Supplemental Figures.docx]

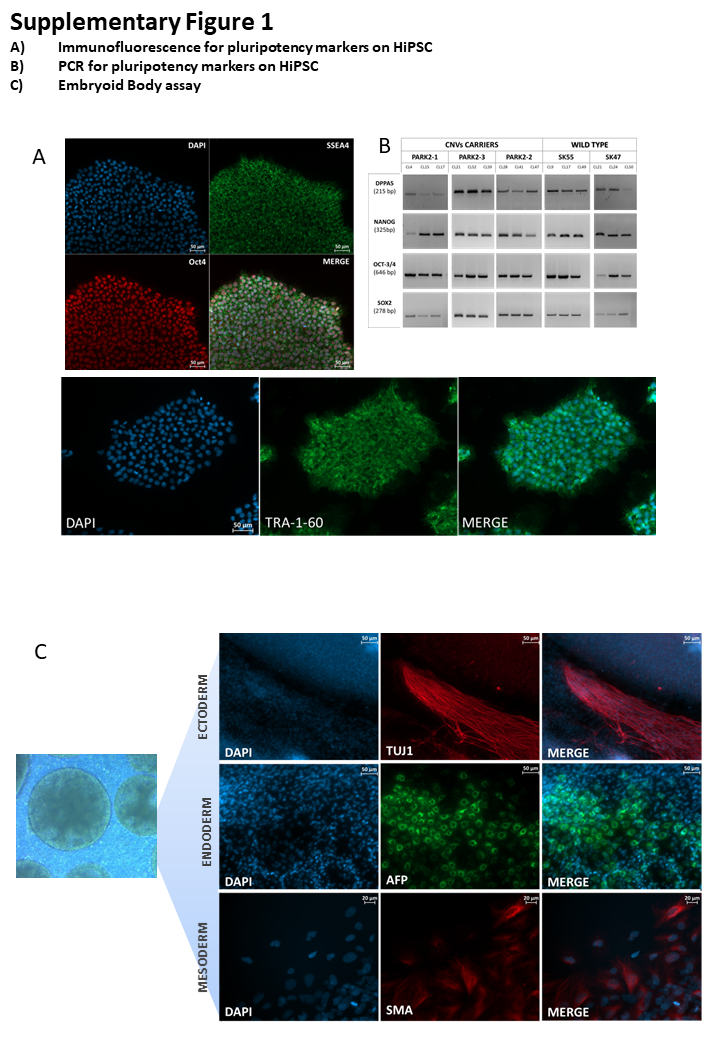


**Figure S1.** Pluripotency tests and markers.


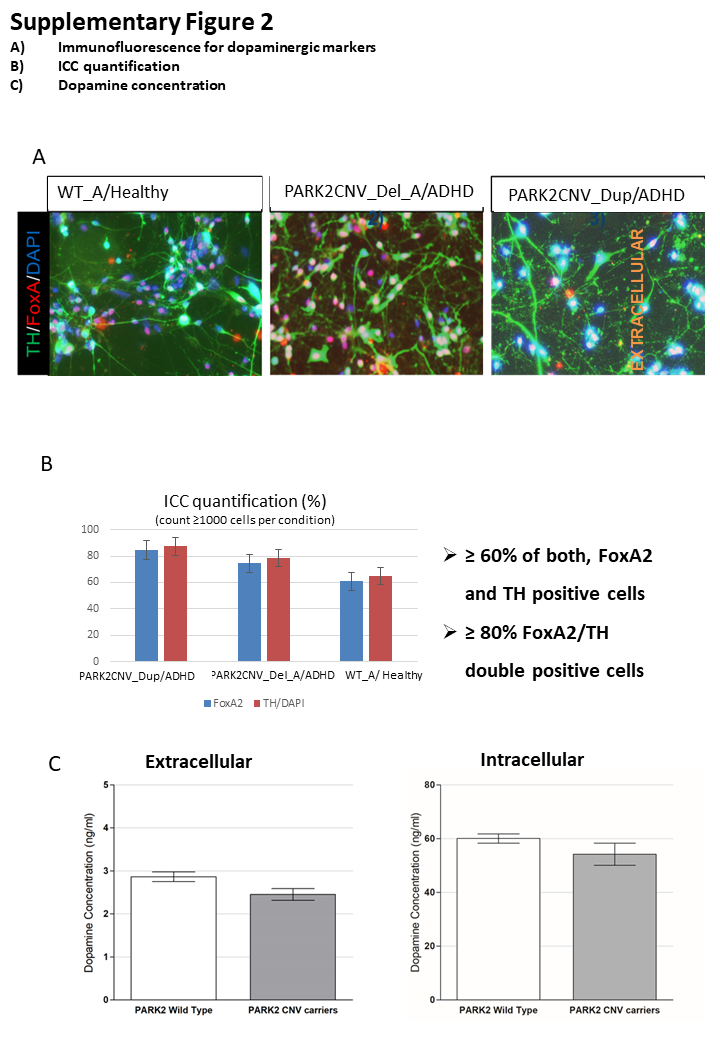


**Figure S2.** Dopaminergic markers of differentiated neuronal cells.


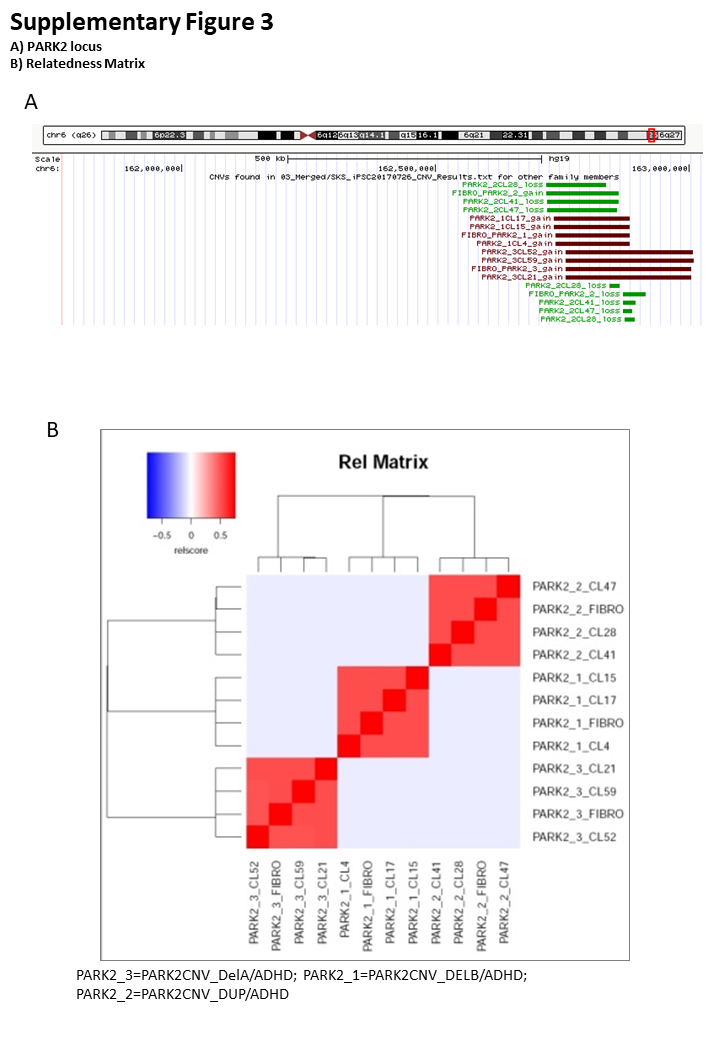


**Figure S3.** PARK2 gene locus and relatedness matrix of DNA from generated hiPSC cells and fibroblasts of PARK2 CNV carrier.


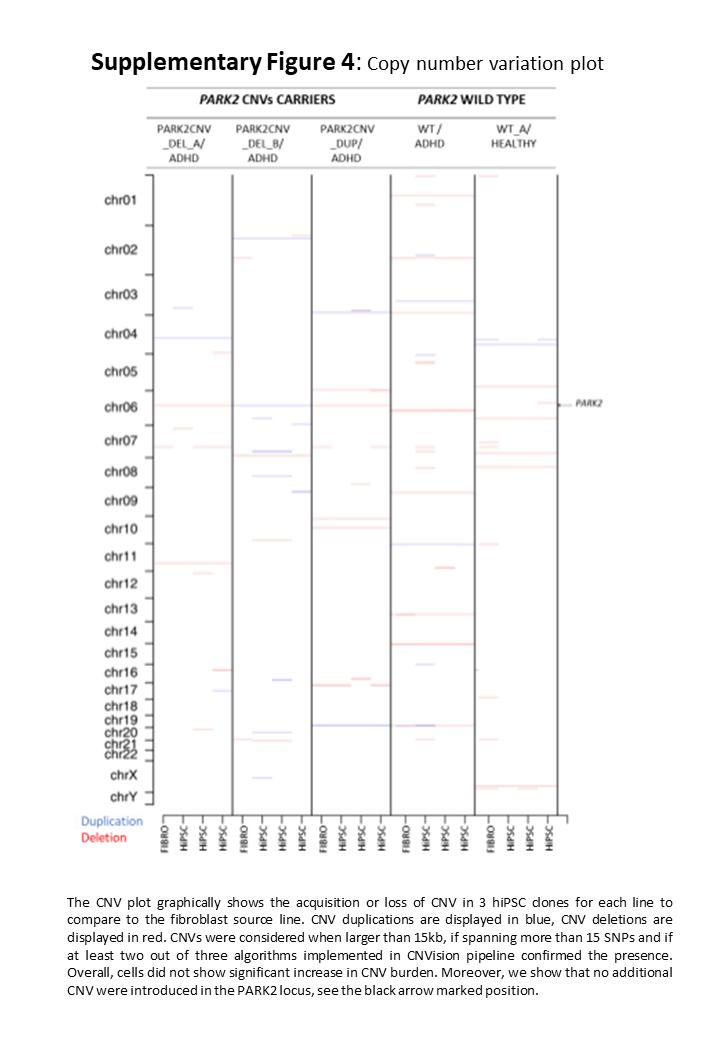


**Figure S4.** Copy number variation plot of all generated hiPSC lines,.


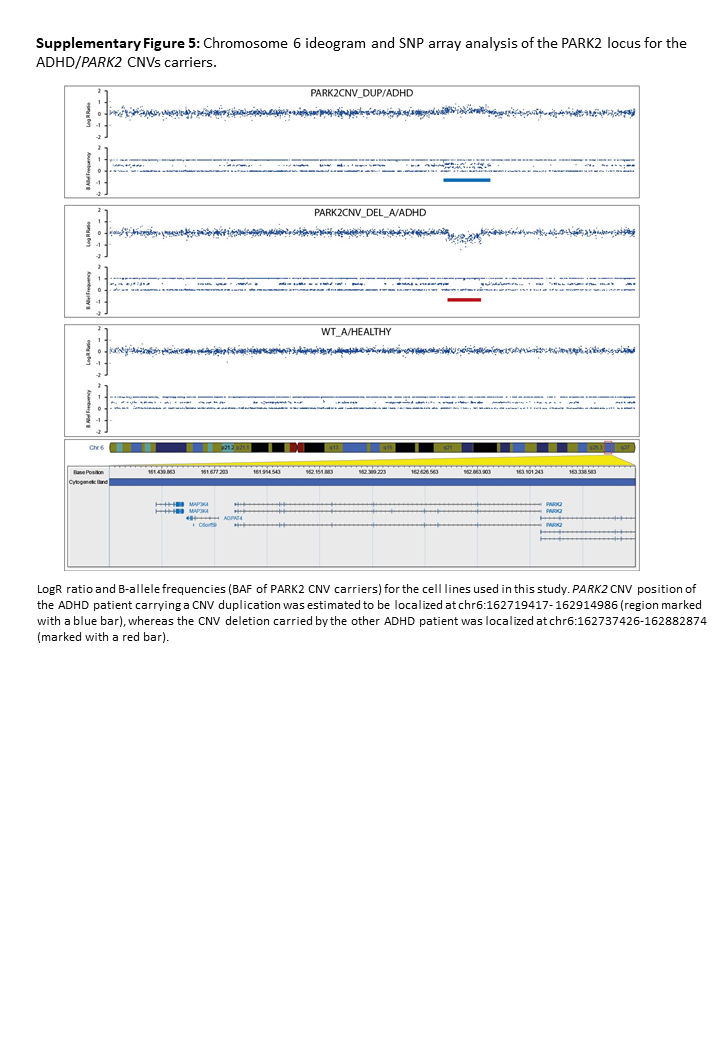


**Figure S5.** Chromosome 6 ideogram and SNP array analysis of the PARK2 locus.


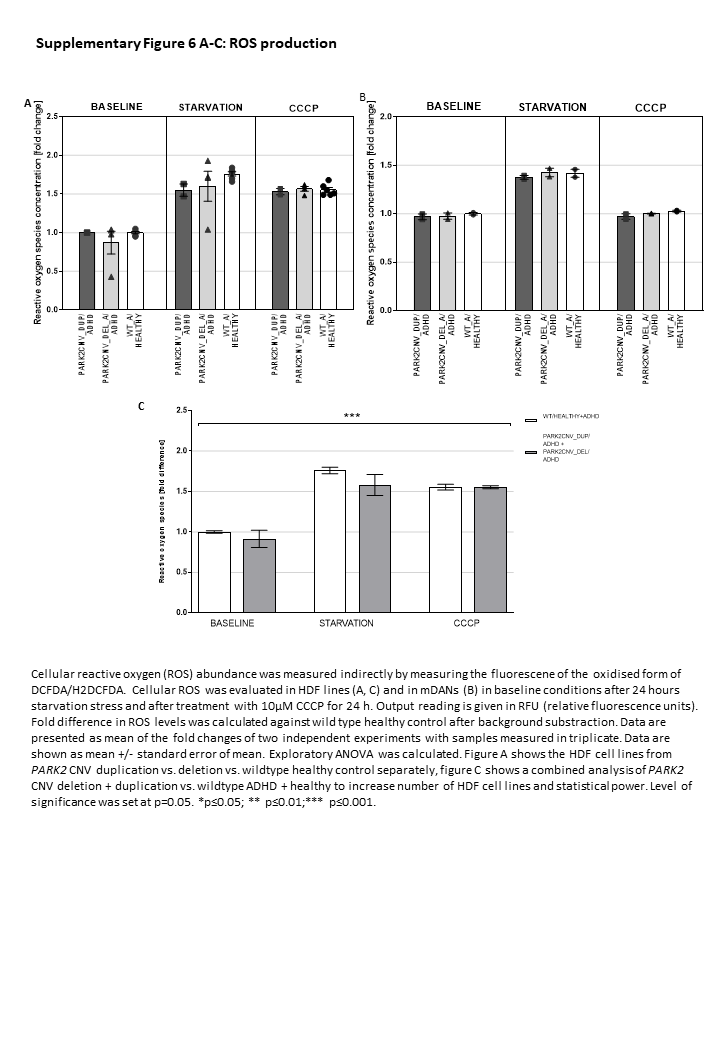


**Figure S6.** ROS production assay.
