# Supplementary material for: Energy Metabolism Disturbances in Cell Models of PARK2 CNV Carriers with ADHD
Source: J Clin Med. 2020 Dec 18;9(12):4092. doi: 10.3390/jcm9124092 (PMC7766864; doi:10.3390/jcm9124092)
Supplement: Supplementary file 1 [file jcm-09-04092-s001.zip › jcm-991097-supplementary/jcm-991097-Supplemental Material 1.docx]

**Supplementary Material 1:** Material and methods Details.

Neuropsychiatric assessment and genotyping

Patients were recruited in 2013 at the Department of Psychiatry, Psychosomatics and Psychotherapy, University of Würzburg, Germany within a previously published sample [1] (see Supplementary Table 1). ADHD in adulthood was independently diagnosed by two trained psychiatrists (SKS and AR) following the DSM-IV diagnostic criteria, whereas childhood symptomatology was assessed retrospectively by the German version of the WURS-k [2]. The healthy controls were recruited among hospital staff and did not report a history of psychiatric disorders, acute or chronic infectious diseases, or severe neurological or internal diseases. Participants were also neurologically examined for early signs of Parkinson's disease by Unified Parkinson's Disease Rating Scale (UPDRS), Non-Motor Symptom assessment scale for Parkinson’s disease (PD NMS) and the sniffing test to assess olfactory function. Additionally, *Substantia nigra* volume was assessed by ultrasound and IQ was measured using the MWT-B (Multiple-Choice Vocabulary Intelligence Test, *Mehrfachwahlwortschatztest*, verbal intelligence) [3].

Skin biopsies and fibroblast primary cultures

Skin biopsies were taken by medically-trained personnel (SKS) under local anaesthesia (Scandicain, AstraZeneca, Wedel, Germany) using a skin puncher (3 mm2). Biopsies were incubated overnight with 1 µg/ml gentamicin in D-PBS with Ca++ and Mg+ (Life Technologies, Carlsbad, USA). After removal of fat tissue, biopsies were washed three times and incubated for 16 hours in Dispase II solution (2.4 U/ml; PAN Biotech, Aidenbach, Germany) at 4 °C. After washing, the epidermis layer was removed and the dermis was incubated with collagenase (SERVA Germany, Heidelberg, Germany) for 45 min at 37 °C. The dermis was then plated and maintained in Dulbecco's modified eagle's medium (DMEM high glucose/pyruvate), 10% fetal bovine serum (FBS, Gibco®, ThermoFisher Scientific, Waltham, USA) and gentamicin 1µl/ml (Life Technologies, Carlsbad, USA) until the growth of fibroblasts. Dermis sample was then removed, and fibroblast cells were detached by Trypsin/EDTA incubation (PAN Biotech Aidenbach, Germany) incubation and replated in adherent cell culture wells (ThermoFisher Scientific, Carlsbad, USA), and medium was changed every other day with DMEM plus 10% FBS.

hiPSCs generation and pluripotency assays

CytoTune-IPs 2.0 Sendai Reprogramming Kit (Invitrogen, Carlsbad, USA) was used to reprogram the fibroblasts into hiPSCs following the manufacturer’s protocol. Incubation with the viral vector (vector multiplicity of infection (MOI): KOS = 9, cMyc = 9, Klf4 = 6) was performed for 24h in DMEM, supplemented with 10% FBS, 1% MEM Non-Essential Amino Acids, 55µM 2-mercaptoethanol (Life Technologies, Carlsbad, Germany). Medium was replaced every other day. According to the manufacturer’s protocol, cells were seeded on irradiated mouse embryonic fibroblasts (Amsbio, Abingdon, UK) at day seven (different densities: 2.5 × 10^4^, 5x10^4^ , 1x10^5^) and media was switched to Knock Out DMEM/F12 (Life Technologies, Carlsbad, Germany), supplemented with 20% Knockout Serum Replacement, 1% MEM Non-Essential Amino Acids 100x, 55µM 2-Mercaptoethanol (Life Technologies, Carlsbad, Germany) and 1% penicillin/streptomycin (Sigma-Aldrich, Taufkirchen, Germany). Three to four weeks after transduction early clones emerged. Single colonies were manually picked and transferred onto new culture ware (Matrigel Corning Matrix-coated plates, Corning, Corning, USA) for clonal expansion. At this stage, hiPSCs were fed daily with mTeSR1 (StemCell Technologies, Vancouver, Canada) and cultured on Matrigel matrix-coated plates (Corning, Corning, USA). One day before reaching 90% confluency, cells were split as single cell suspension 1:2 using ReLeSR (StemCell Technologies, Vancouver, Canada).

For each line 3 single clones (derived from single colonies) were selected for expansion and *bona fide* hiPSC characterization was carried out after passage 12. All lines were routinely tested for mycoplasma and acholeplasma contamination using the Venor GeM Mycoplasma Detection Kit (Merck Millipore, Darmstadt, Germany) according to the manufacturer’s instructions.

The formation of three-dimensional aggregates (embryoid bodies; EBs) was achieved by seeding 1.0x10^7^ hiPSCS for 24 h as single cell suspension on an AggreWell 400Ex plate (Stem Cell Technologies, Vancouver, Canada) in AggreWell medium, supplemented with 10 µM Y-27632 (Stem Cell Technologies, Vancouver, Canada). EBs were grown in suspension culture on low-adhesion plates (Corning, Corning, USA) and fed every other day. From day 11 on, cultures were fed with DMEM/F-12, supplemented with 10% Knockout Serum Replacement, 1% MEM Non-Essential Amino Acids 100x and 0.1 mM 2-mercaptoethanol (Life Technologies, Carlsbad, USA). After two weeks, EBs were seeded on Matrigel -coated coverslips and cultured for another six days (for pluripotency tests, see Supplementary Figure 1 and Supplementary Table 2 and 3).

Differentiation of hiPSCs into neurons with a midbrain dopamine like phenotype

Differentiated cells were maintained on Geltrex-coated plates in Neurobasal/B27/L-glutamine medium (Thermofisher Scientific, Waltham, USA) supplemented with BDNF (20 ng/ml) (Cell guidance Systems, Cambridge, GB), GDNF (20 ng/ml) (Cell guidance Systems, Cambridge, GB), L-ascorbic acid (LAAP, 220 µM) (Sigma Aldrich, Taufkirchen, Germany), TGFß-III (1 ng/ml) (Peprotech, Hamburg, Germany), dibutyryl-cAMP (0.5 mM) (Enzo, Lörrach, Germany) and DAPT (10 µM) (Axon Medchem LLC, Reston, USA).

Immunofluorescence and mitostaining

For immunofluorescence assays cells were grown on Matrigel-coated coverslips, fixed with 4% paraformaldehyde for 20 min, permeabilized with 0.2% Triton-X-100 for 15 min and blocked with 3% BSA (all Sigma Aldrich, Taufkirchen, Germany) for 30 min at room temperature. Primary antibodies (Supplementary material Table 3) were incubated overnight at 4 °C; secondary antibodies were incubated for 1 hour at RT. Coverslips were mounted on glass slides with ProLong Diamond Antifade Mountant with DAPI (Life Technologies, Carlsbad, USA). Slides were imaged with a fluorescence microscope with ApoTome function (Zeiss, Oberkochen, Germany). For the analysis of mitochondrial network morphology, fibroblast cells were grown on coverslips and mitochondria were stained with 400 nM MitoTracker® Red CMXRos (ThermoFisher Scientific, Waltham, USA) for 30 min. Cells were fixed in 4% PFA for 15 min at room temperature and nuclei stained with NucBlue Fixed Cell Stain (ThermoFisher Scientific Waltham, USA) during the last wash step and incubated for 5 min. Coverslips were mounted on glass slides with ProLong Diamond Antifade Mountant (Life Technologies, Carlsbad, USA) for imaging. 15 different (blinded for the genotype selection) selected fibroblasts for each line/condition from two independent experiments were captured with Zeiss Axio Observer.Z1 microscope with ApoTome function (Zeiss, Oberkochen, Germany) and analysed by a semi-automated analysis with Fiji ImageJ software as described elsewhere [4]. Shape descriptors considered were aspect ratio (AR): major_axis/minor_axis and Form factor (FF): [perimeter2/(4π × area)], both of which were calculated for each mitochondrion present in the cells. Mean values for each analysed cell were used for statistical analysis. The values were obtained by analysing 15 cells per line/condition from two independent experiments. The form factor describes the morphological properties of the mitochondrial network (mitochondrial branching). It is calculated as: [perimeter2/(4π × area)] and small values are indicative of a more fragmented-dotted network, whereas higher values describe a more tubular, chain-like network. The aspect ratio mainly describes the shape of the mitochondria and is calculated as the ratio between the major axis and the minor axis of the ellipse equivalent to the object. A value of 1 describes a perfect circular shape whereas increased values describe a more elongated shape.

RNA extraction, Two steps Reverse Transcription PCR (RT-PCR) and Quantitative RT-PCR (RT-qPCR)

RNA was isolated using RNeasy-Plus Mini Kit (Qiagen, Hilden, Germany) according to manufacturer’s instructions. RNA quality and absence of gDNA contamination was assessed using the Standard Sensitivity RNA Analysis Kit with Fragment Analyzer (Advanced Analytical, Agilent, Santa Clara, USA). RNA with RQN >9 and a 28S/26S ratio above 1.5 was used for further applications. A total of 500ng of RNA was converted into cDNA by RT-iScript cDNA Synthesis Kit (Bio Rad, Hercules, USA). Given the low gene expression of *PARK2* in fibroblast, samples subsequently used for *PARK2* gene expression evaluation were pre-amplified. Pre-amplification of the target genes was performed with TaqMan PreAmp Master Mix Kit (ThermoFisher Scientific, Waltham, USA) according to the manufacturer protocol. TaqMan PreAmp Pool was obtained by combining 10 µl of each probe (*PARK2*, *B2M*, *YWHAZ*, *POLR2A*, *SDHA*) with TE buffer as suggested by the manufacturer. The preamplification reaction was performed Mastercycler nexus X2 (Eppendorf, Hamburg, Germany) after the manufacturer’s instructions. Predesigned primer and probe sets used in the assay for the target gene *PARK2* is reported in Supplementary Table 2. Data analysis was performed as described above. Quantitative RT-PCR (RT-qPCR) Primers were designed to be Intron spanning with NCBI Primer-Blast with the size of the respective intron being greater than 1 kbp (sequence on Supplementary material table 2). The total reaction volume was 10µl in a formulation of 1 µl cDNA, 3 µl RNase free water, 1 µl primer mix (10 pmol/µl) 5 µl FastStart Essential DNA (Roche, Basel, Switzerland). Plates were read in LightCycler 2.0 (Lifescience-Roche, Basel, Switzerland). For each primer a standard curve was created using quadruplets for each dilution and the absence of primer dimers or unspecific products was confirmed by Melting curve analysis with LightCycler 480 SW 1.5.1 (Lifescience-Roche, Basel, Switzerland). Primer efficiency and linearity of the amplification were evaluated by Genex ver6 (Biomet, Warsaw, USA). *ALAS1, TBP, HRPT1* and *SDHA* were used as reference genes. The best reference genes to be used for normalization were chosen according the lowest M-values obtained using the geNorm feature of Genex ver6 (Biomet, Warsaw, USA).To obtain the fold difference, Delta Ct where averaged to the percentage of total gene expression and log2 transformed.

Protein concentration

Whole cell lysates were obtained by incubation with Pierce Lysis Buffer supplemented with 10 µl/ml Halt Protease and Phosphatase Inhibitor Cocktail (ThermoFisher Scientific, Waltham, USA) for 10 min on ice. For the fibroblast cultures, cell pellet was additionally homogenized using pestle homogenizer (Schuett-Biotec, Göttingen, Germany). Protein concentration from whole cell lysated was determined with ADV02 assay (Cytoskeletron Inc, Denver, USA) and absorbance measured at 600 nm wavelength using Infinite M200 PRO microplate reader (Tecan, Männedorf, Switzerland). The values were measured in duplicates, averaged and corrected by subtraction of the background value. Protein concentration was extrapolated by interpolation with a standard curve performed with known serial dilutions of BSA (Sigma Aldrich, St. Louis, USA).

PARK2 protein levels where measured with Human Parkin SimpleStep (Enzyme-linked Immunosorbent Assay) ELISA Kit (Abcam, Cambridge, UK) following the manufacturer instructions. 1000 µg/ml of total protein extract was loaded in each well. Endpoint reading of Optical Density (OD) at 450 nm was performed on Infinite M200 PRO microplate reader (Tecan, Männedorf, Switzerland). For every assay, 8 standards with known concentration of human PARK2 obtained from extract of HEK293T cells overexpressing human parkin was included. Results from HDF lines were obtained from two independent experiments with samples measured in duplicate, results from mDA neurons were obtained from one experiment with samples measured in duplicate.

Molecular karyotyping

Genomic DNA from cell lines was extracted with the DNeasy kit (Quiagen, Hilden, Germany) and analysed on an Illumina Infinium Omni2.5-8 bead array at the Institute of Human Genetics, LIFE&BRAIN, University of Bonn. SNP calling was performed using the GenomeStudio 2.0 software and the GenTrain-Algorithm 2.0 with a GenCall-Threshold of 0.2. All samples had a SNP-call rate above 99%. CNVs were called implementing the CNVision pipeline based on the LogR and BAF scores exported from the FinalReport exports from GenomeStudio. CNVs were considered to be valid if spanning more than 15 SNPs, if larger than 15kb and if at least two out of three algorithms implemented in the CNVision pipeline confirmed the presence. CNV annotation was performed using the BAMotate algorithm. For visualization, regions of 2Mb are shown and coloured (red = deletion, blue = duplication) if any CNV was present within the respective region. SNP-based relatedness-analysis to confirm lineage identity was performed on the pedigree export format (Genome studio plink plug-in) using plink v 1.9 (<https://www.cog-genomics.org/plink/>) and the IBD function --genome function. Visualization is based on the PI_HAT score (see Figures S3 and S4).

ATP production, oxygen consumption and ROS production

All assays were conducted on black-sided 96-well plates with clear bottoms (Greiner Bio one, Frickenhausen, Germany) and in DMEM without phenol red (Thermofisher Scientific, Waltham, USA). Before plating, cells were counted using an EVE Automated Cell Counter (NanoEnTek, Seoul, Korea). After the assay, the number of cells was confirmed using the CyQUANT assay (ThermoFisher Scientific, Waltham, USA) and the values were corrected for the actual cell number. Plates were read using a Plate reader Infinite 200 PRO (Tecan, Männedorf, Switzerland). ATP production was measured using the ATPlite Luminescence Assay System (PerkinElmer, Walluf, Germany) according to manufacturer’s instructions. ROS production was measured using the DCFDA/H2DCFDA - Cellular Reactive Oxygen Species Detection Assay Kit (Abcam, Cambridge, GB), according to a protocol suggested by the manufacturer for 24h of treatment. Tert butylhydroperoxide (TBHP) treated cells in all conditions were added to the samples as positive control for the assay. After blank correction, fold difference was calculated in relation to the WT genotype under baseline conditions. Basal extracellular oxygen consumption rate (OCR) was measured using the Extracellular O2 Consumption Assay (Abcam, Cambridge, Germany) as suggested by the manufacturer, and results were reported as relative fluorescence units (RFU). All experiments in all cell lines were done in technical triplicates or duplicates and the mean of the triplicates or duplicates is shown.

References

1. Jarick, I.; Volckmar, A.-L.; Pütter, C.; Pechlivanis, S.; Nguyen, T.T.; Dauvermann, M.R.; Beck, S.; Albayrak, Ö., Scherag, S.; Gilsbach, S.; et al. Genome-wide analysis of rare copy number variations reveals PARK2 as a candidate gene for attention-deficit/hyperactivity disorder. *Molecular Psychiatry*. **2014**, *19*, 115–121. DOI:https://doi.org/10.1038/mp.2012.161.
2. Retz-Junginger, P.; Retz, W.; Blocher, D.; Weijers, H.G.; Trott, G.E.; Wender, P.H., Rössler, M. Wender Utah rating scale. The short-version for the assessment of the attention-deficit hyperactivity disorder in adults. *Nervenarzt*. **2002**, *73*, 830–838.
3. Lehrl, S.; Triebig, G; Fischer, B. Multiple choice vocabulary test MWT as a valid and short test to estimate premorbid intelligence. *Acta Neurologica Scandinavica*. **1995**, *91*, 335–345. DOI:https://doi.org/10.1111/j.1600-0404.1995.tb07018.x.
4. Burbulla, L.F.; Kruger, R. The use of primary human fibroblasts for monitoring mitochondrial phenotypes in the field of Parkinson’s disease. *J. Vis. Exp.* **2012**, *68*, 4228. DOI:10.3791/4228.
