# Supplementary material for: Energy Metabolism Disturbances in Cell Models of PARK2 CNV Carriers with ADHD
Source: J Clin Med. 2020 Dec 18;9(12):4092. doi: 10.3390/jcm9124092 (PMC7766864; doi:10.3390/jcm9124092)
Supplement: Supplementary file 1 [file jcm-09-04092-s001.zip › jcm-991097-supplementary/jcm-991097-Supplemental Tables.docx]

**Table S1:** Phenotypic and demographic data.

| *PARK2* CNV Genotype | Disorder | Cell Line Code | Sex | Ethnicity | Age | IQ | MDS UPDRS Part III, Motor Score | Sniffing Sticks | S. Nigra Right cm^2^ | S. Nigra Left cm^2^ | HDF | hiPSC | mDAN |
| --- | --- | --- | --- | --- | --- | --- | --- | --- | --- | --- | --- | --- | --- |
| Deletion | ADHD | PARK2CNV_DEL_A/ADHD | female | Caucasian | 43 | 118 | 1 | 10 | 0.08 | 0.15 | Yes | Yes | Yes |
| Duplication | ADHD | PARK2CNV_DUP/ADHD | female | Caucasian | 28 | 104 | 2 | 11 | 0.14 | 0.10 | Yes | Yes | Yes |
| Deletion | ADHD | PARK2CNV_DEL_B/ADHD | male | Caucasian | 47 | 130 | 2 | 10 | 0.11 | 0.09 | Yes | Yes | No |
| Wildtype | ADHD | WT/ADHD | female | Caucasian | 30 | 107 | 0 | 11 | 0.10 | 0.07 | Yes | Yes | No |
| Wildtype | Control | WT_B/HEALTHY | female | Caucasian | 27 | 118 | 1 | 11 | 0.13 | 0.14 | Yes | No | No |
| Wildtype | Control | WT_A/HEALTHY | female | Caucasian | 39 | 136 | 0 | 10 | 0.10 | 0.15 | Yes | Yes | Yes |

ADHD = attention-deficit-/hyperactivity disorder; IQ = intelligence quotient; MDS-UPDRS = Movement disorder society sponsored revision of the unified Parkinsons’s disease rating scale; HDF = Human dermal fibroblasts; hiPSC = human induced pluripotent stem cells, Mdan = midbrain dopaminergic neurons.

**Table S2:** Primer sequences for PCR and qRT PCR.

|  | Target | Forward | Reverse |
| --- | --- | --- | --- |
| (RT)-PCR | Hs_SOX2 | AACCAGCGCATGGACAGTTA | GACTTGACCACCGAACCCAT |
|  | Hs_NANOG | ACCAGTCCCAAAGGCAAACA | AAAGGCTGGGGTAGGTAGGT |
|  | Hs_OCT4 | GTTGATCCTCGGACCTGGCTA | GGTTGCCTCTCACTCGGTTCT |
|  | Hs_DPPA5 | CGGCTGCTGAAAGCCATTTT | AGTTTGAGCATCCCTCGCTC |
|  | SeV | GGATCACTAGGTGATATCGAGC | ACCAGACAAGAGTTTAAGAGATATGTATC |
|  | c-Myc | TAACTGACTAGCAGGCTTGTCG | TCCACATACAGTCCTGGATGATGATG |
|  | Hs_PARK2 | CCAAACCGGATGAGTGGTGA | TGCGATCAGGTGCAAAGCTA |
|  | Hs_HPRT1 | TGCTTTCCTTGGTCAGGCAGT | TCCAACACTTCGTGGGGTCC |
|  | Hs_SDHA | AACATCGGAACTGCGACTC | CTTCTTGCAACACGCTTCCC |
|  | Hs_ALAS1 | CGGGATGGAGTCATGCCAAA | ATCAGAGAACTCGTGCTGGC |
|  | Hs_TBP | GAGTTCCAGCGCAAGGGTTT | GGGGTCAGTCCAGTGCCATA |

**Table S3:** Primary and secondary antibodies used for immuncytochemistry.

|  | Antibody and Host | Dilution | Company Cat # and RRID |
| --- | --- | --- | --- |
| Primary antibodies | Rabbit anti-OCT4 | 1:500 | Thermo Fisher Scientific Cat# 710788, RRID: AB_2633097 |
|  | Mouse anti-SSEA4 | 1:200 | Thermo Fisher Scientific Cat# MA1-021, AB_2536687 |
|  | Mouse anti-TRA-1-60 | 1:100 | Novus Cat# NB100-730, RRID:AB_10001809 |
|  | Rabbit anti-TUJ1 | 1:700 | Thermo Fisher Scientific Cat# A25532, RRID:AB_2651003 |
|  | Mouse anti-AFP | 1:700 | Thermo Fisher Scientific Cat# A25530, RRID:AB_2651004 |
|  | Mouse anti-SMA | 1:200 | Thermo Fisher Scientific Cat# A25531, RRID:AB_2651005 |
|  | Mitotracker Red |  |  |
| Secondary antibodies | Alexa Fluor 594 donkey anti-rabbit | 1:250 | Thermo Fisher Scientific Cat# R37119, RRID:AB_2556547 |
|  | Alexa Fluor 488 goat anti-mouse IgG3 | 1:250 | Thermo Fisher Scientific Cat# A-21151, RRID:AB_2535784 |
|  | Alexa Fluor 488 Goat anti-mouse IgM | 1:250 | Thermo Fisher Scientific Cat# A-21042, RRID:AB_2535711 |
|  | Alexa Fluor 488 goat anti-mouse IgG1 | 1:250 | Thermo Fisher Scientific Cat# A25536, RRID:AB_2651011 |
|  | Alexa Fluor 555 goat anti-mouse IgG2a | 1:250 | Thermo Fisher Scientific Cat# A25533, RRID:AB_2651012 |
|  | Alexa Fluor 647 donkey anti-rabbit | 1:250 | Thermo Fisher Scientific Cat# A25535, RRID:AB_2651010 |

**Table S4:** PARK 2 gene expression in HDF.

| HDF Cell Lines | Baseline  (±SEM) | Starvation  (±SEM) | *p*-Value |
| --- | --- | --- | --- |
| *PARK2*CNV_DUP/ADHD | 1.25 | 1.95 | 0.085 |
| *PARK2*CNV_DEL/ADHD | 0.44 ± 0.06 | 1.34 ± 0.85 |  |
| WT HEALTHY+ADHD_ | 2.36 ± 0.98 | 2.11 ± 0.44 |  |

Displayed are normalised expression values of relative mRNA expression measured by qRT PCR. HDF cells lines were used and PARK2CNV_DEL_A/ADHD and PARK2CNV_DEL_B/ADHD as well WT_A/HEALTHY as WT_B/HEALTHY and WT/ADHD were analysed as groups to investigate if there was a difference in gene expression in *PARK2* duplication vs. *PARK2* deletion vs. wildtype. Data are shown as mean ± standard deviation. Explorative ANOVA was calculated. Level of significance was set at *p* = 0.05.
